# Accuracy of compressed sensing for left ventricular mass and volumes

**DOI:** 10.1186/1532-429X-16-S1-P381

**Published:** 2014-01-16

**Authors:** Suzanne Lydiard, Andreas Greiser, Michaela Schmidt, Michael O Zenge, Mariappan S Nadar, Alistair Young, Brett R Cowan

**Affiliations:** 1 Auckland MRI Research Group, University of Auckland, Auckland, New Zealand; 2 Healthcare Sector, Siemens AG, Erlangen, Germany; 3 Siemens Corporate Research, Siemens AG, Princeton, New Jersey, USA; 4 Centre for Advanced MRI, University of Auckland, Auckland, New Zealand

## Background

While SSFP CMR is the gold standard for assessing left ventricular (LV) function, it requires a regular cardiac rhythm and frequent breath-holds and not all patients with cardiovascular disease are able to achieve this. It is known that Compressed Sensing (CS) greatly reduces data acquisition time however its accuracy for LV volume and mass is currently unknown. This study compares ventricular function measurements by CS to those obtained from standard SSFP cines.

## Methods

Twenty healthy human subjects (9 male, 40 ± 14 years) underwent LV SSFP imaging on a MAGNETOM Skyra 3T scanner (Siemens, Germany). Three sequences were acquired (i) gold standard fully sampled SSFP (FULL) and two 2D prototype sequences featuring CS reconstruction and regularisation in space and time with acceleration factors (ii) R = 4 (R4) or (iii) R = 9.2 (R9.2). 5-8 short axis slices (thickness 6 mm, slice gap 9 mm) and three long axis slices (4-,3-,2-chamber), FOV = 260-340 mm, were acquired for each sequence. FULL images were acquired over 14 heart-beats with TE = 1.54 ms, α = 51°, 25 frames, matrix 256×256 and iPAT factor 2. R4 images were acquired over 4 beats with TE = 1.29 ms, α = 41°, 21 frames, matrix 192×143 with iPAT. R9.2 images were acquired over 2 beats (one dummy beat for steady state preparation, thereby representing 'realtime' acquisition) with TE = 1.27 ms, α = 42°, 19frames, matrix 192×129. Images were reconstructed on-line using a non-linear iterative CS method with k-t regularisation derived from a SENSE type reconstruction [1]. Ventricular volume and mass were measured by two analysts blinded to image type using CIM Version 7 and averaged.

## Results

R4 produced comparable end-diastolic volume (EDV) and ejection fraction (EF) results but there were significant differences in end-systolic volume (ESV) and LV mass (LVM). R9.2 results were comparable for ESV but significantly different for EDV, EF and LVM. While statistically significant, these differences were small and consistent, and similar to other acceleration techniques [2,3]. To quantify the clinical significance of these results, effect sizes (ES) were calculated, with only EF showing any significance difference for R4 and R9.2.

## Conclusions

These in-vivo results suggest that CS may be used to efficiently assess ventricular function in normal subjects. Testing on pathological cases is required to further support this conclusion. Further optimization of the sampling pattern and image reconstruction parameters is also likely to improve image quality and reconstruction times.

## Funding

Siemens Medical Systems.

**Table 1:** LV functional parameters for the FULL acquisition, and difference from this for each accelerated sequence.

	EDV mean ± std (mL)	ESV mean ± std (mL)	EF mean ± std (%)	Mass mean ± std (g)
FULL	146.0 ± 31.6	56.9 ± 16.3	61.4 ± 4.0	113.9 ± 30.3

FULL minus R4	-1.2 ± 5.5	-2.4 ± 3.4 *	1.4 ± 1.9	-7.2 ± 7.1*

FULL minus R9.2	7.3 ± 5.8**	-2.7 ± 4.8	4.1 ± 2.6**	-4.7 ± 6.2**

p values represent repeated measures Bonferroni corrected ANOVA, * represents 0.05 < p < 0.01,** represents p < 0.01

**Figure 1:**
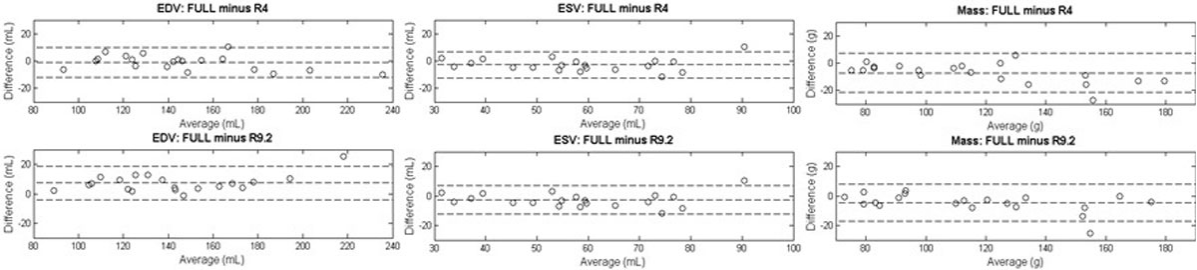
**Bland-Altman analysis of VF comparing FULL and R4 (top row) or FULL and R9.2 (bottom row) sequences**.
